# A single institution experience of combined modality management of extra skeletal Ewings sarcoma

**DOI:** 10.1186/1477-7819-5-3

**Published:** 2007-01-11

**Authors:** Ramachandran Venkitaraman, Mathew K George, S Ganapathy Ramanan, TG Sagar

**Affiliations:** 1 Division of Clinical Oncology, Royal Marsden Hospital, Sutton, Surrey, SM2 5PT, UK; 2 Division of Medical Oncology, Royal Darwin Hopsital, Casuarina, Australia; 3 Division of Medical Oncology, Cancer Institute(WIA), Adyar, Chennai, 400036, India

## Abstract

**Background:**

Extraskeletal Ewings sarcoma are rare tumors for which there is no consensus on optimal management.

**Methods:**

A retrospective review of the clinical features, treatment and outcome of patients with extraskeletal Ewings sarcoma who reported to a single institution between January 1992 – December 2003 is reported.

**Results:**

A total of 19 patients with extraskeletal Ewings sarcoma were identified. Of these, 4 patients had metastatic disease at presentation and 15 patients with non-metastatic disease received combined modality treatment with primary combination chemotherapy followed by local treatment with radiotherapy or surgery. Disease free survival and overall survival for patients with non metastatic disease after combined modality treatment were 60% and 30% respectively. The significant predictors for prolonged disease free survival and overall survival were high haemoglobin(p = 0.002), low lactate dehydrogenase (p = 0.028), chemotherapy with Vincristine, Adriamycin, Cyclophosphamide, Ifosfamide and Etoposide regime (p = 0.008) and complete response to chemotherapy (p = 0.001).

**Conclusion:**

Aggressive combination chemotherapy followed by complete surgery or radiotherapy to a dose of more than 50 Gy is essential to confer optimal outcome for patients with extraskeletal ewings sarcoma.

## Background

Ewing's family of tumors arise primarily from bones and rarely are of extraskeletal origin. Considering the rarity and varied presentation of extraskeletal Ewings sarcoma (ESES), no definite recommendation regarding optimal treatment has been defined. The principles of management of extraskeletal Ewings tumors have been extrapolated from experience from treating Ewings sarcoma of bony origin or primitive peripheral neurectodermal tumors (PPNET). Commonly ESES patients are offered combined modality treatment as for Ewing's sarcoma (ES) of the same site and stage, though some studies have suggested that extraosseous primaries may have a poorer prognosis[1]. We are presenting a single institution experience of management of this uncommon tumour along with a review of relevant literature.

## Patients and methods

We conducted a retrospective review of the case records of patients who had histologically proven Ewing's family of tumors of extraskeletal origin who reported to the Cancer Institute between January 1992 – December 2003. Criteria for inclusion in the study were a) Soft tissue tumours without any bone involvement on CT scan, b) Histology suggestive of Ewings family of tumors with Homer – Wright rosettes and neurofilaments The standard patient evaluation consisted of history and clinical examination, complete blood counts, biochemistry, computed tomography (CT) scan of the primary site, CT scan thorax, radionuclide bone scan and bone marrow biopsy. All patients had cytochemical studies with periodic acid shiff stain(PAS) and immunohistochemical stains with vimentin, Neuron Specific Enolase(NSE), CD 99(mic2), S100 protein to confirm neurectodermal origin and staining with epithelial membrane antigen, cytokeratin, leucocyte common antigen, desmin and muscle actin to rule out other malignant tumors. Cytogenetic and electron microscopic studies were conducted if samples were available. The demographic profile, clinical characteristics, pathology, treatment and outcome were analysed. Patient characteristics which were continuous variables were dichotomised over the median value for ease of statistical analysis. Disease free survival and overall survival were plotted by the Kaplan Meier method and significance of differences between variables to predict for survival were analysed by log rank test, with a p value < 0.05 considered significant. Multivariate analysis of predictors of survival were analysed by Cox proportional hazards model. Statistical analysis was conducted with SPSS14 (SPSS Inc, Chicago, IL)

### Demographics

The study population consisted of 19 patients, 12 male and 7 female. The median age was 21 (range 3 – 73). The primary sites of disease were pelvis in three, retroperitoneum in two, paraspinal region in three, extremities in seven and thorax in four patients (table 1).

**Table 1:** Patient characteristics

Total number	19
Median Age	23 years (range 3 – 73)

Male	12
Female	7

Site of disease :	
Thorax	4
Upper extremity	3
Retroperitoneum	2
Paraspinal	3
Pelvis	3
Lower extremity	4

Maximum tumour diameter in cm	10.5 cm (6 – 20)
Ewings Sarcoma	8
(CD99 +, PAS+) PPNET (NSE+)	7

Eight patients were CD99, PAS and vimentin positive and were categorised as Ewings sarcoma. Eight patients were NSE and/or S100 protein positive and were categorised as PPNET. Three patients were classified as malignant small round cell tumour on morphology

Four patients presented with metastatic disease, one each in the lungs, bone, bone marrow and scalp. These patients received palliative radiotherapy and palliative chemotherapy. All four patients had a short partial response to treatment, had progressive disease soon after and a short survival.

### Treatment

The 15 patients with non metastatic disease received initial combination chemotherapy, which was scheduled to continue for eighteen months. The three different chemotherapy combinations used were VAC (Vincristine, Actinomycin D, Cyclophosphamide), VACA (Vincristine, Actinomycin D, Cyclophosphamide, Adriamycin) and VAC alternating with IE (Ifosfamide and Etoposide). Five patients each received the three chemotherapy regimes. The median number of cycles received was 8 cycles (range 1–18)

One patient defaulted treatment and was lost to follow-up. Of the 14 patients who completed combination chemotherapy, 8 patients had a complete response, two had a partial response and 4 had progressive disease.

Four of the five patients who received VAC chemotherapy, had progressive disease while on treatment. Of the 5 patients who received VACA chemotherapy, 3 patients had a complete response, one had progressive disease, while one patient defaulted. All five patients who had VAC/IE achieved a complete response and continue to be disease free.

A total of 11 patients were assessed for local treatment. Three patients who progressed on VAC chemotherapy and one patient who defaulted on VACA were not available for assessment. Of these, 8 patients had a complete response or a very good partial response, while 3 patients had partial response to chemotherapy. Three patients who had a less than complete response underwent surgery. One patient underwent amputation and two patients had a complete resection of post chemotherapy residue. All three specimens showed viable tumor with areas of necrosis on pathological examination.

The patients who had a radiological complete response or a good partial response to chemotherapy were offered external beam radiotherapy. Six patients received 40 Gy and three patients received more than 50 Gray.

## Results

The median follow-up was 12 months. Seven patients remain on follow-up and are disease free. Three patients developed bone metastasis. One patient with paraspinal tumour had a late relapse at the site of initial disease. None of the patients who had a complete response to chemotherapy had a late metastatic relapse. Three of the six patients who had 40 Gy relapsed on follow-up, while all the three patients who received more than 50 Gy remain disease free.

**Figure 1:**
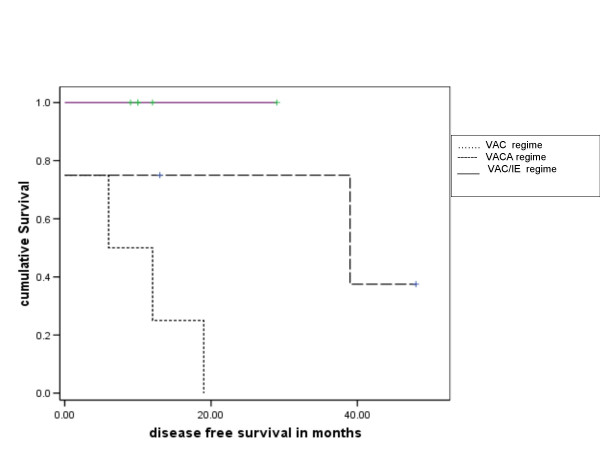
Kaplan Meier plot of overall survival in patients according to chemotherapy regime received [Group 0 – metastatic disease (nochemotherapy); Group 1 – VAC chemotherapy; Group 2 – VACA chemotherapy; Group 3 – VAC/IE chemotherapy (V – Vincristine, A – Actinomycin D, C – cyclophosphamide, A – Adriamycin, I – Ifosfamide, E – etoposide)] significance of differences by log rank test p = 0.008

**Figure 2:**
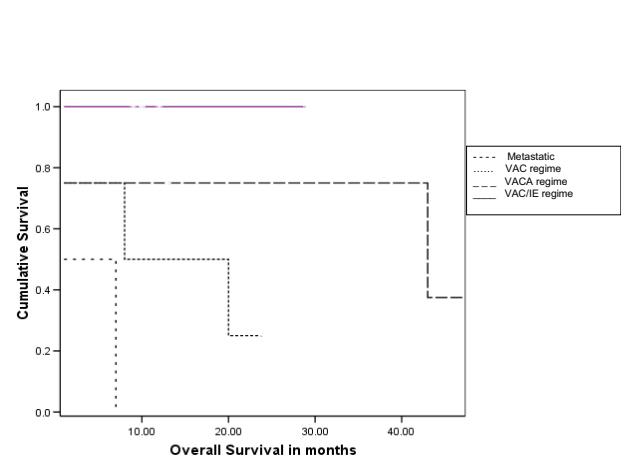
Kaplan Meier plot of disease free survival in patients who received chemotherapy [Group 0 – no chemotherapy; Group 2 – VAC chemotherapy; Group 3 – VACA chemotherapy; Group4 – VAC/IE chemotherapy (V – Vincristine, A – Actinomycin D, C – cyclophosphamide, A – Adriamycin, I – Ifosfamide, E – etoposide)] significance of differences by log rank test p = 0.026

The 3 year and 5 year disease free survival for all patients were 38% and 19% respectively, and overall survival 47% and 24% respectively. Of the 15 treated patients (excluding the patients who presented with metastatic disease), the 3 y and 5 yr overall survival were 60 % and 30% respectively (Figure 1 and 2).

The significant predictors of prolonged disease free survival on univariate analysis were high Haemoglobin(p = 0.002), low Lactate dehydrogenase (p = 0.028), chemotherapy with VAC/IE regime (p = 0.008) and complete response to chemotherapy (p = 0.001), while male sex (p = 0.238), site (p = 0.464), size > 10.5 cm (p = 0.7), older age (p = 0.341), Ewings Vs PPNET (p = 0.169), low serum alkaline phosphatase (p = 0.098), radiotherapy dose ≥ 50 Gy (p = 0.164) were not statistically significant.

The significant predictors for prolonged overall survival on univariate analysis were similar with high haemoglobin (p = 0.013), low lactate dehydrogenase (p = 0.022), chemotherapy regime with VAC/IE regime (p = 0.026) and complete response to chemotherapy (p = 0.001), while male sex(p = 0.482), site (p = 0.832), size > 10.5 cm (0.76), older age (p = 0.166), Ewings Vs PPNET (p = 0.169), low serum alkaline phosphatase (p = 0.388), radiotherapy dose ≥ 50 Gy (p = 0.065) were not statistically significant. None of the variables were found to independently predict for disease free or overall survival on multivariate analysis.

## Discussion

Our series of nineteen patients with ESES varied in the age, site and stage of presentation. The diagnosis of extraskeletal ewings sarcoma requires a combination of clinical features, radiology, pathology, immunohistochemistry and molecular techniques. The following criteria are proposed for the diagnosis of primary ESES: (i) no evidence of bony involvement on magnetic resonance imaging (ii) no evidence of increased uptake in bone or periosteum adjacent to the tumour on static isotope bone scan images (iii) a small round cell tumour with no differentiating features on light microscopy, immunochemistry or electron microscopy and (iv) demonstration of cytoplasmic glycogen[2]. Light microscopy and electron microscopic features of ESES and ES of skeletal origin have been shown to be identical [3-5]. Results from previous studies suggest that PPNET of bone or soft tissue origin are identical and that ES (or ESES) and PPNET are histogenetically related, representing different stages of cell differentiation[6]. We could not detect any significant differences in response to chemotherapy or difference in survival between tumours which on histopathology were classified as either Ewings sarcoma or as PPNET.

Baldini *et al*., have shown in his series of patients with Ewings Sarcoma that non-skeletal primary may be an unfavourable predictor for survival, the other adverse predictors being metastasis at diagnosis and older age[1]. Ahmad *et al*., in 24 patients found that younger age and complete resection predict for a better survival in ESES [7]. Rud *et al*., in 42 cases noted decreased survival with pelvic primary, incomplete resections, and presence of metastatic disease[8].

In our series, age, site of presentation and tumour size did not reach significance. None of the patients with metastatic disease at presentation had a good response to chemotherapy and all these patients had a dismal survival. Low level of haemoglobin and high lactate dehydrogenase adversely predicted for survival, probably reflecting initial high tumour burden.

We had used the sequence of initial systemic chemotherapy followed by local treatment taking into consideration the natural history of ES/PPNET where majority of failures are distant. We found that aggressiveness of chemotherapy was a significant factor predicting survival. Patients with non metastatic disease were treated with primary chemotherapy, the regime depending on the period of presentation. Patients who presented between 1992 – 1996 received VAC regime, while those who presented between 1996–2000 received VACA regime and those who presented later received VAC/IE, which mirrored the evolution of chemotherapy regimes during the study period. Though the period of follow-up might be shorter in patients who received VAC/IE, the results were significantly superior to three or four drug combinations. Patients who had a complete response to chemotherapy had prolonged survival suggesting that initial aggressive initial treatment may be beneficial.

The results from published studies regarding optimal mode of local treatment has been inconsistent. Rud *et al*., and Covelli *et al*., have suggested that surgery has may have a more important role in ESES than in skeletal ES and that complete resection predicts a favourable survival[8,9]. Kinsella *et al*., have suggested that combined modality therapy consisting of high-dose local irradiation and vincristine, actinomycin D and cyclophosphamide chemotherapy may be sufficient for ESES and that radical surgery may be unnecessary[10]. Complete surgery if feasible may be a better option considering the late side effects of high dose radiotherapy especially second malignancy. We found a dose effect for radiotherapy with a dose of 50 Gy required for adequate control, though not statistically significant. The role of adjuvant local radiotherapy after complete resection is still inconclusive too has been shown to improve increased survival[8]

From our experience all patients who received chemotherapy with five drugs and had complete surgery or radiotherapy to a dose of more than 50 Gy were alive with no evidence of disease.

## Conclusion

Our experience in managing extraskeletal Ewings sarcoma underlines the importance of aggressive combined modality treatment to confer optimal outcome for this uncommon tumour.

## Competing interests

The author(s) declare that they have no competing interests.

## Authors' contributions

The patients in the study were clinically managed by **SGR **and **TGS**. Data collection was carried out by **RV **and **MKG**. Data analysis was conducted by **RV **and **MKG**. The draft of the manuscript was made by **RV **and **MKG**, which was critically reviewedand approved by **SGR **and **TGS**. All the authors have read and approved the final mansucript.
